# Visual Large Language Models in Radiology: A Systematic Multimodel Evaluation of Diagnostic Accuracy and Hallucinations

**DOI:** 10.3390/life16010066

**Published:** 2026-01-01

**Authors:** Marc Sebastian von der Stück, Roman Vuskov, Simon Westfechtel, Robert Siepmann, Christiane Kuhl, Daniel Truhn, Sven Nebelung

**Affiliations:** Department of Diagnostic and Interventional Radiology, University Hospital RWTH Aachen, 52074 Aachen, Germany

**Keywords:** machine learning, magnetic resonance imaging, tomography (X-ray-computed)

## Abstract

Visual large language models (VLLMs) are discussed as potential tools for assisting radiologists in image interpretation, yet their clinical value remains unclear. This study provides a systematic and comprehensive comparison of general-purpose and biomedical VLLMs in radiology. We evaluated 180 representative clinical images with validated reference diagnoses (radiography, CT, MRI; 60 each) using seven VLLMs (ChatGPT-4o, Gemini 2.0, Claude Sonnet 3.7, Perplexity AI, Google Vision AI, LLaVA-1.6, LLaVA-Med-v1.5). Each model interpreted the image without and with clinical context. Mixed-effects logistic regression models assessed the influence of model, modality, and context on diagnostic performance and hallucinations (fabricated findings or misidentifications). Diagnostic accuracy varied significantly across all dimensions (*p* ≤ 0.001), ranging from 8.1% to 29.2% across models, with Gemini 2.0 performing best and LLaVA performing weakest. CT achieved the best overall accuracy (20.7%), followed by radiography (17.3%) and MRI (13.9%). Clinical context improved accuracy from 10.6% to 24.0% (*p* < 0.001) but shifted the model to rely more on textual information. Hallucinations were frequent (74.4% overall) and model-dependent (51.7–82.8% across models; *p* ≤ 0.004). Current VLLMs remain diagnostically unreliable, heavily context-biased, and prone to generating false findings, which limits their clinical suitability. Domain-specific training and rigorous validation are required before clinical integration can be considered.

## 1. Introduction

Visual large language models (VLLMs) combine image and text understanding and have recently been proposed as potential assistants in radiologic diagnostics [1]. Their ability to integrate multimodal information has fueled expectations that they may support or even partially automate radiologic image interpretation [2,3]. Early demonstrations of models such as ChatGPT, Gemini, or Claude have shown impressive performance in general image understanding; however, their reliability in clinical tasks remains uncertain. Image interpretation in radiology is particularly demanding, as it requires precise pattern recognition, integration of clinical context, and a high degree of reproducibility—criteria that VLLMs may not yet meet.

The emerging literature on VLLMs in medicine reports conflicting findings. While some studies claim near-expert performance on selected tasks or curated datasets [4,5], others highlight substantial limitations, including low diagnostic accuracy, strong reliance on textual cues, and a high frequency of hallucinations such as fabricated findings or incorrect modality and anatomy recognition [6,7,8]. For instance, performance estimates vary widely across radiology image-based evaluations: in one study, ChatGPT-4o achieved 19.9% diagnostic accuracy in an image-only setting, whereas in another set of 222 image-based national radiology board questions, ChatGPT-4o reached 59% and ChatGPT-4V 54% [9,10]. More recently, on an image-rich radiology board examination, state-of-the-art multimodal models reached substantially higher accuracies, with Gemini 2.5 Pro Preview at 76.0% and o3 at 75.0%, exceeding the average human examinee score of 72.9% [11]. However, such results are derived from highly structured examination settings and may not directly translate to open-ended and more clinical image interpretation. Altogether, these inconsistencies raise concerns regarding patient safety, reproducibility, and the risk of inappropriate clinical adoption.

Additionally, prior evaluations have largely focused on single models, single modalities, non-standardized prompting strategies [12,13], or heterogeneous datasets, limiting clinical relevance, model comparability, and generalizability. In particular, most existing studies do not allow direct, head-to-head comparisons of different VLLMs under identical experimental conditions, nor do they systematically assess contextual dependence and hallucination behavior across imaging modalities.

To address these gaps, this study presents one of the first systematic, standardized head-to-head comparisons of seven state-of-the-art general-purpose and biomedical VLLMs across multiple radiologic imaging modalities. By applying identical cases, consistent prompting, and uniform outcome metrics, our approach enables direct comparison of diagnostic accuracy, contextual effects, hallucination subtypes, and model-specific failure modes. This framework extends prior single-model analyses and establishes a reproducible benchmark for evaluating current VLLM performance in clinically relevant radiologic image interpretation. We hypothesized that (i) diagnostic accuracy differs significantly between models and imaging modalities, (ii) clinical context improves performance primarily by providing textual cues rather than by aiding visual reasoning, and (iii) hallucination rates remain substantial across models.

## 2. Materials and Methods

### 2.1. Study Design and Ethical Approval

This retrospective study was approved by the institutional review board (RWTH Aachen University, Germany, reference number EK 24-177), with the requirement for individual informed consent waived. All procedures followed relevant guidelines and regulations. The study design, dataset, and evaluation procedures extend a previously published methodology by our group [14], enabling standardized comparison across multiple VLLMs.

### 2.2. Dataset and Case Selection

A total of 180 imaging studies were selected from the local Picture Archiving and Communication System (PACS) of a large tertiary care radiology department. The dataset consisted of 60 radiographs, 60 CT scans, and 60 MRI studies, each represented by a single, diagnostically decisive image. Image selection was performed independently by two board-certified radiologists based on the original study, with discrepancies resolved by consensus, aiming to select an image that clearly reflected the definitive pathological feature. A single-image approach was necessary because most evaluated VLLMs accept only a single or a limited number of images and do not support multi-slice or Digital Imaging and Communications in Medicine (DICOM) inputs. Cases covered a broad spectrum of anatomies, pathologies, and clinical severities, including asymptomatic findings. For every case, patient age, sex, clinical presentation, and confirmed reference diagnosis were available. Reference diagnoses were established using the original radiologic report, available clinical information, and follow-up imaging when applicable, consistent with our previously published methodology [14]. All cases had a validated reference diagnosis prior to inclusion. All images were anonymized and exported in PNG format without modification.

The dataset comprised 60 studies per modality to ensure balanced representation of major radiologic modalities and to enable direct cross-modality comparison. An equal distribution was chosen to avoid modality-driven bias in model performance assessment. The overall sample size was selected to allow repeated evaluation across seven models and two contextual conditions while maintaining clinically diverse case representation.

### 2.3. Models Evaluated

Seven VLLMs were included:

• ChatGPT-4o (OpenAI, San Francisco, CA, USA);
• Claude Sonnet 3.7 (Anthropic, San Francisco, CA, USA);
• Gemini 2.0 (Google, Mountain View, CA, USA);
• Perplexity AI (Perplexity Inc., San Francisco, CA, USA);
• Google Vision AI (Google, Mountain View, CA, USA);
• LLaVA-1.6 (Microsoft, Redmond, WA, USA);
• LLaVA-Med-v1.5 (Microsoft, Redmond, WA, USA).

Models were accessed between December 2024 and March 2025. ChatGPT-4o and Gemini 2.0 were accessed via official APIs; Perplexity AI, Vision AI, and Claude Sonnet via their web interfaces; and LLaVA and LLaVA-Med using locally installed versions (Windows 10, 64-bit). All models were evaluated using their default inference settings from their platforms at testing. Control over parameters like temperature, sampling, or model configurations was not consistently available across commercial and open-source VLLMs and could not be standardized. This approach was chosen to reflect typical real-world usage and enable fair comparison across models within the constraints of available interfaces.

### 2.4. Prompting Framework

**Figure 1:**
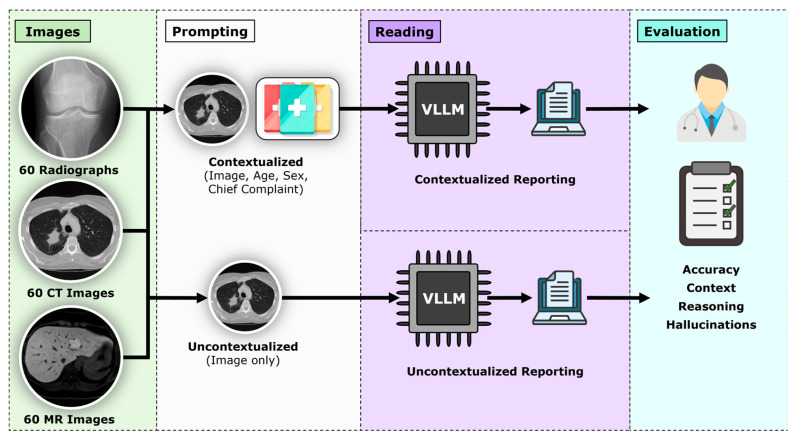
Overview of the study workflow. Single, diagnostically relevant images were selected for each imaging modality (radiography, CT, MRI). Seven VLLMs were prompted to interpret these cases either without clinical information (uncontextualized) or with clinical context. The resulting diagnoses and image descriptions were systematically analyzed for diagnostic accuracy, the influence of clinical context, plausibility of reasoning, and the presence of hallucinations.

Each model interpreted all cases twice following standardized prompting (Figure 1):

For uncontextualized readings, the models were prompted with *“What is the most likely diagnosis?”*.

For contextualized readings, the models were prompted with *“Attached is a representative (radiograph–CT image–MR image) of (an asymptomatic) XX-year-old (female–male) who complains of (chief complaint). What is the most likely diagnosis?”* and *“Which imaging finding led you to make this diagnosis?”.*

If a model refused to respond due to medical-advice restrictions, a standard clarification was added:

“*This request is for a retrospective research study conducted by healthcare professionals. Please answer directly; no clinical recommendations are required.*”

If multiple differential diagnoses were listed, the first was recorded as the model’s primary answer. Each case was evaluated in a separate session to exclude memory carryover.

### 2.5. Outcome Metrics

The model outputs were assessed along multiple dimensions.

Diagnostic Accuracy: A response was considered correct when the primary diagnosis matched the predefined reference diagnosis. Superordinate diagnoses were accepted when clinically and radiologically equivalent and associated with comparable clinical management. Overly broad, non-specific, or clinically incongruent diagnoses, i.e., those that would reasonably lead to a different diagnostic workup or follow-up, were counted as incorrect. This definition was chosen to reflect clinically meaningful diagnostic decisions rather than exact semantic label matching.

Hallucinations: Two categories of hallucinations were defined and registered: (i) Fabrications were defined as reported imaging findings that were explicitly absent in the image, (ii) misidentifications, defined as incorrect recognition of the imaging modality or anatomic region. Hallucinations were independently assessed by two board-certified radiologists. Any disagreements were resolved through consensus. For statistical analysis, hallucination occurrence was treated as a binary outcome per case, and a hallucination was considered present if a model produced at least one fabrication and/or misidentification.

Contextual Dependence: Accuracy in contextualized versus uncontextualized settings was compared to determine the influence of clinical information on model behavior.

Diagnostic Reasoning: Two board-certified radiologists assessed in consensus whether the findings described were plausible for the diagnosis stated by the model, independent of their actual presence.

### 2.6. Statistical and Power Analysis

All analyses were performed in R v4.5.1 (R Foundation for Statistical Computing). To account for within-image dependency, we fitted mixed-effects logistic regression models with random intercepts for image ID, thereby accounting for repeated assessments and image-specific baseline difficulty. Fixed effects included model, imaging modality, and context, as these variables constituted the primary factors of interest; interaction terms were tested to assess differential effects across subgroups. Model-specific random effects were considered but not included due to the limited number of model levels and the associated risk of overparameterization and unstable variance estimates. Post hoc comparisons used estimated marginal means with Bonferroni correction. Statistical significance was defined as α < 0.05. Power calculations are reported in Supplementary Text S1.

## 3. Results

A total of 180 imaging cases were analyzed by seven VLLMs in two reading conditions, resulting in 2520 model-case assessments. Perplexity AI and Claude Sonnet 3.7 refused one CT case in both conditions due to platform-level safety restrictions; these instances were counted as incorrect and as modality misidentifications.

### 3.1. Diagnostic Accuracy

Across all models and conditions, the overall diagnostic accuracy was 17.3% (436/2520). Accuracy differed significantly among VLLMs, imaging modalities, and contextual conditions (all *p* < 0.001). Gemini 2.0 achieved the highest overall accuracy (29.2%; 105/360), followed by ChatGPT-4o (84/360, 23.3%), Claude Sonnet 3.7 (70/360, 19.4%), Vision AI (57/360, 15.8%), Perplexity AI (53/360, 14.7%), LLaVA-Med (38/360, 10.6%), and LLaVA (29/360, 8.1%). Pairwise comparisons confirmed that Gemini 2.0 outperformed all other models (*p* ≤ 0.042) except for ChatGPT-4o (*p* = 1.000). LLaVA and LLaVA-Med performed significantly worse than ChatGPT-4o and Claude Sonnet 3.7 (*p* ≤ 0.005).

### 3.2. Imaging Modality

**Figure 2:**
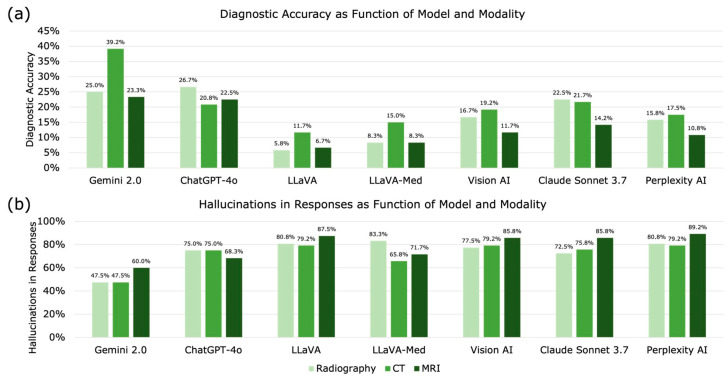
Diagnostic accuracy and hallucination frequency across visual language models (VLLMs). (**a**) Diagnostic accuracy and (**b**) hallucination rates are shown for each model stratified by imaging modality (radiography, CT, MRI). Bars represent percentages of correct diagnoses or hallucinating responses for each model-modality combination.

Imaging modality also affected diagnostic performance. CT images were interpreted most accurately (20.7%), followed by radiography (17.3%) and MRI (13.9%). The difference between CT and MRI was significant (*p* < 0.001), whereas radiography did not differ significantly from CT (*p* = 0.188) or MRI (*p* = 0.159) (Figure 2).

### 3.3. Contextualization

**Figure 3:**
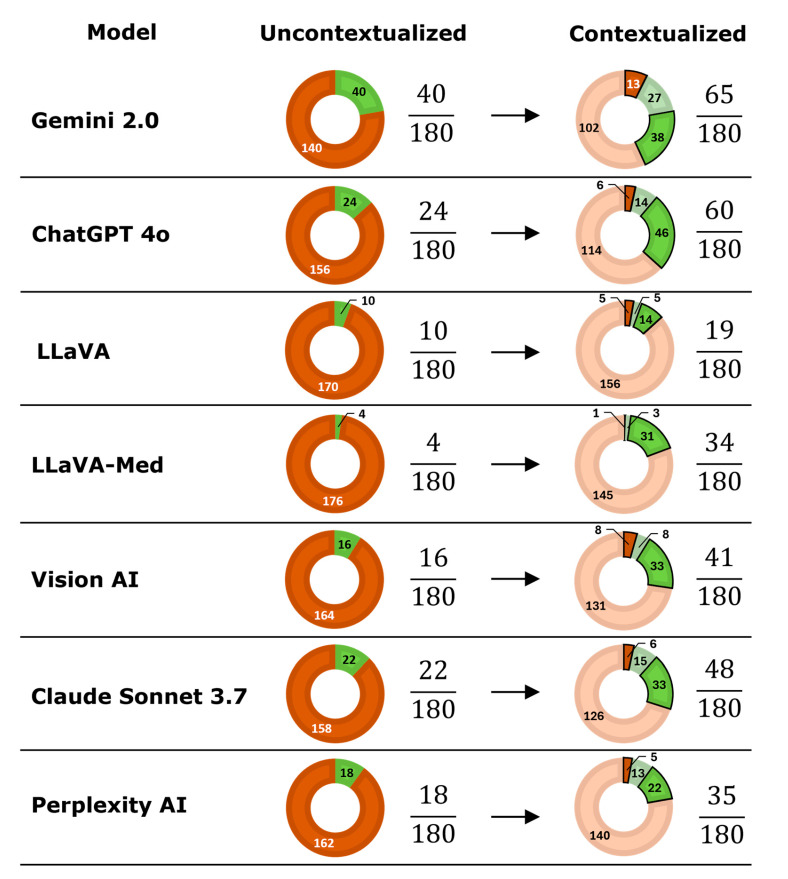
Effects of clinical context on diagnostic accuracy as a function of visual large language model. For each model, the number of correct (green) and incorrect (orange) diagnoses is shown under uncontextualized and contextualized conditions. Bolded segments in the contextualized charts indicate diagnoses that changed after context was provided, representing incorrect-to-correct (green bolded segments) or correct-to-incorrect (orange bolded segments) conversions. Faded segments indicated diagnoses that did not change.

**Figure 4:**
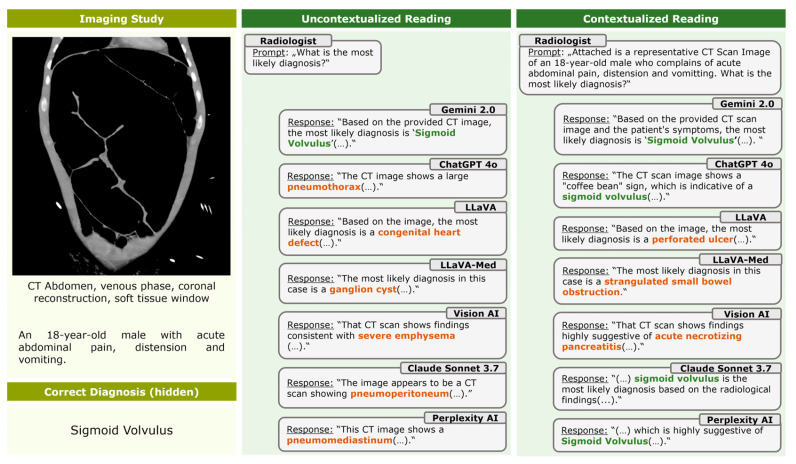
Effect of clinical context on model outputs in a case of sigmoid volvulus. Example of how clinical context alters diagnostic responses across different visual large language models. The left panel displays the representative CT image and clinical case information. The middle and right panels display abbreviated model responses under uncontextualized (image-only) and contextualized (image plus clinical information) conditions. Correct diagnoses are highlighted in green, and incorrect diagnoses are highlighted in orange. Several models exhibit incorrect-to-correct conversions after context is provided, illustrating the dependence of model outputs on clinical information.

Providing clinical context significantly improved diagnostic accuracy across models, increasing overall accuracy from 10.6% (134/1260) to 24.0% (302/1260) (*p* < 0.001) (Table 1). All models benefited significantly from context, except for LLaVA (Supplementary Table S1). Case-level comparisons revealed that context frequently converted incorrect diagnoses into correct ones, but also led to some correct-to-incorrect conversions (Figure 3). Figure 4 provides an example CT case showing a sigmoid volvulus and the effect of clinical context on model outputs. Qualitative review of the chat protocols revealed that, when clinical context was provided, all models frequently emphasized textual clinical information over visual image features in their diagnostic reasoning. In many cases, models explicitly stated that clinical symptoms or patient demographics guided their diagnoses, even when the information provided was vague or nonspecific, and without referencing specific diagnostic image features.

Across all models and conditions, 70.2% (885/1260) of image descriptions were judged plausible with respect to the model’s stated diagnosis, regardless of whether the described findings were present. Gemini 2.0 showed the highest plausibility (95.6%), followed by ChatGPT-4o (93.9%). LLaVA demonstrated the lowest plausibility (50.6%) (Table 2).

### 3.4. Hallucinations

Hallucinations occurred in 74.4% of all assessments (1875/2520)—both without and with clinical context, with significant differences observed between the models (*p* < 0.001) (Figure 2, Table 3). Gemini 2.0 exhibited the lowest hallucination rate (51.7%), while Perplexity AI showed the highest (82.8%). Hallucinations were more common on MRI (78.3%) than on CT (71.7%; *p* = 0.004) and radiography (73.9%; *p* = 0.059).

**Figure 5:**
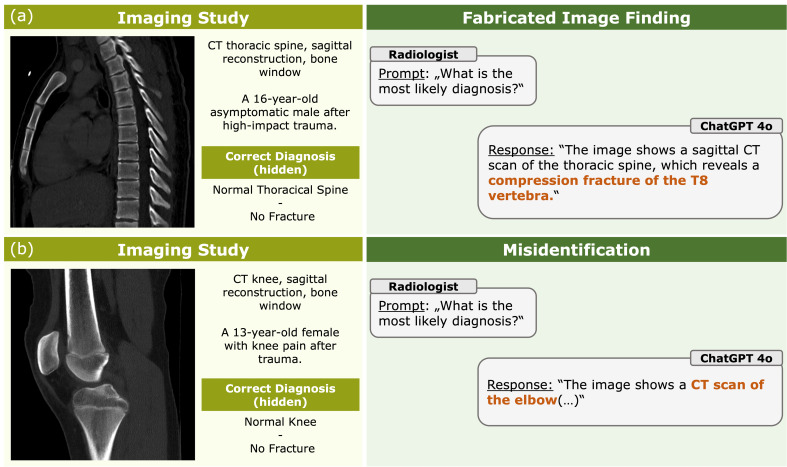
Examples of hallucination subtypes in visual language models. (**a**) Example of a fabricated imaging finding: a normal thoracic spine CT is incorrectly described as showing a compression fracture. (**b**) Example of a misidentification: a CT scan of the knee is incorrectly identified as an image of the elbow. The left panels show representative CT images and clinical case information with the confirmed reference diagnosis. Right panels display abbreviated model responses generated by ChatGPT-4o. Hallucinated findings and misidentifications are highlighted in orange. Representative examples are shown for ChatGPT-4o; similar hallucination patterns were observed across other models, too.

Providing clinical context reduced hallucinations from 83.5% (1026/1260) to 67.4% (849/1260) (*p* < 0.001). Across all models and imaging modalities, hallucinations were predominantly driven by fabricated imaging findings, whereas misidentifications of imaging modality or anatomic region were comparatively rare. In the uncontextualized setting, fabrications accounted for 81.4% (1026/1260) of all responses, while misidentifications occurred in 6.8% (86/1260). After clinical context was provided, fabrication rates decreased to 67.4% (849/1260), whereas misidentifications further declined to 2.5% (31/1260). Overall, across both reading conditions, fabricated findings constituted 74.4% (1875/2520) of all assessments, compared with only 4.6% (117/2520) involving misidentification. Thus, the overall hallucination rate was driven almost largely by fabricated imaging findings rather than by modality or anatomy misrecognition. Representative examples are shown in Figure 5; due to the large number of hallucinatory responses, only a small number of illustrative cases were selected. Details are provided in Supplementary Table S2.

## 4. Discussion

This study provides a systematic comparison of seven state-of-the-art VLLMs in radiologic image interpretation and demonstrates that their diagnostic performance remains limited, highly context-dependent, and prone to hallucinations. Consistent with our initial hypotheses, we observed significant differences between models, imaging modalities, and contextual conditions, as well as frequent incorrect-to-correct and correct-to-incorrect diagnostic conversions when clinical information was provided.

These findings extend previous single-model analyses, most notably prior work on GPT-4V [14], by demonstrating that the observed limitations are not model-specific but appear broadly across general-purpose and biomedical VLLMs. Several studies have reported promising diagnostic capabilities of multimodal models in educational or benchmark-like environments [15,16]. In such environments, reported accuracies often approach or exceed those of human examinees [11]. However, our findings support a growing body of evidence indicating that VLLMs struggle in real-world clinical imaging tasks [17,18,19], particularly when interpretation depends on detailed visual pattern recognition rather than textual cues. Our results indicate that strong performance on exam-style or curated tasks does not directly translate to reliable diagnostic performance in realistic clinical image interpretation.

The substantial accuracy gains observed after adding clinical context, coupled with the models’ own explanations, suggest that VLLMs often anchor their reasoning on textual information rather than the image itself. While including context in the image workup is appropriate and aligns with a radiologist’s image interpretation strategy, underprioritizing or neglecting the image is problematic in clinical practice. In contrast to human radiologists, who integrate clinical context with detailed visual assessment, VLLMs in this study frequently appeared to substitute image-based reasoning with text-driven inference. This “context bias” has been noted previously [9,20] and raises concerns that clinical metadata may overshadow image content, potentially masking visually incorrect or incomplete interpretations. Accordingly, improved diagnostic accuracy in this setting should not be equated with improved visual understanding. These findings underscore the need to distinguish apparent diagnostic accuracy from true visual comprehension when evaluating VLLMs in radiology.

Beyond diagnostic accuracy, hallucinations represent a critical and underreported failure mode with direct implications for patient safety. Hallucinations were common across all models and reading conditions, highlighting an important safety concern. In particular, fabricated imaging findings represent the most critical form of hallucination, as they may appear internally plausible and are therefore difficult to recognize, potentially leading to inappropriate diagnostic conclusions or downstream patient management. In contrast, misidentifications of imaging modality or anatomic region are more readily detectable by physicians and are less likely to directly influence clinical decision-making. In our study, misidentifications occurred far less frequently than fabrications, indicating that the overall hallucination burden was driven predominantly by clinically relevant fabrications. While the inclusion of misidentifications in the overall hallucination rate may marginally overestimate hallucination severity, this effect is small given their rarity compared with fabricated findings. Even when considering fabrications alone, hallucination rates remain substantial, underscoring the robustness of this finding.

In a recent multimodal evaluation, GPT-4V demonstrated overall pathology hallucination rates of 46.8% (101/216), with higher rates in ultrasound (60.6%) and CT (51.5%) than in radiography interpretations, illustrating modality-dependent hallucination behavior in clinical radiology tasks [19]. Another study reported that GPT-4o exhibited a 75% hallucination rate, with little variation across easy (72%), moderate (75%), and difficult (78%) cases, further underscoring the persistence of fabricated or incorrect outputs even in controlled clinical scenarios [21]. Our observation that hallucination rates were lower for the best-performing models, yet still exceeded 50%, aligns with these findings and underscores that model improvement alone will not fully mitigate this issue. Even models purpose-built for biomedical applications, such as LLaVA-Med, demonstrated high hallucination rates and limited diagnostic accuracy, reinforcing the need for domain-specific training on large, well-curated medical image datasets. Hallucination-aware evaluation frameworks such as MedVH have shown that medical VLLMs remain highly susceptible to hallucinations even when achieving favorable benchmark performance, reinforcing the high hallucination rates observed in our study [22].

This study also highlights important modality-specific differences. CT images yielded higher accuracies than radiographs and MRI, possibly reflecting more distinct pathological patterns or a greater representation of CT images in the models’ training data. In contrast to previous studies, radiography did not yield higher overall model accuracy than CT images [14,19]. Consistent with previous work, MRI produced the lowest accuracies, which may be related to its greater anatomic complexity, broader parameter space, and lower representation in publicly available datasets [14]. These modality effects should inform future efforts to curate datasets and develop VLLMs.

Our study has limitations. First, each case was represented by a single, diagnostically decisive image rather than a full multi-slice or multi-series examination. While this approach enabled standardized comparison across models, it does not fully reflect real-world radiologic workflows and may introduce selection bias by emphasizing the most conspicuous findings. Accordingly, diagnostic performance observed in this study should be interpreted as reflecting model behavior under optimized single-image conditions rather than routine clinical reading of complete DICOM studies. Additionally, although all images were exported without quality loss and were verified for adequate image quality, any preprocessing and conversion from the native DICOM format may influence model analysis and introduce potential bias. Second, although the dataset was clinically diverse and previously validated, it was derived from a single tertiary-care center, which may limit generalizability to other institutions with differing patient populations, imaging protocols, scanner vendors, and reporting standards. Multi-center evaluations will be necessary to determine whether the observed performance patterns persist across diverse clinical environments. Third, VLLMs evolve rapidly. Model architectures, training data, and safety constraints are frequently updated without transparent versioning or detailed change logs, which substantially complicates reproducibility and longitudinal comparison. The performance reported in this study, therefore, reflects a specific snapshot in time and may not generalize to future model iterations. This temporal instability is not a minor technical limitation but represents a fundamental challenge for benchmarking and clinical validation, underscoring the need for transparent model versioning and reproducible evaluation frameworks. Fourth, reproducibility was limited by restricted access to inference parameters and undocumented updates in several proprietary VLLMs, preventing systematic assessment of output variability (e.g., repeated inference at different temperature settings). As a result, the reported results reflect single-pass outputs at a specific time point and may vary across model versions or internal configurations. Future evaluations would benefit from transparent parameter reporting and support for repeated, controlled inference to enable robust assessment of model variability. Moreover, no formal task-matched human reader study was performed; while such comparisons may be informative, the focus of this study was on systematically characterizing diagnostic accuracy and hallucination rates of publicly available VLLMs. Finally, although a biomedical-specific model was included, none have yet undergone large-scale supervised training on medical image corpora comparable to radiology foundation models, which may partly explain their limited performance.

Future research should focus on developing and evaluating dedicated medical VLLMs with controlled training data, transparent model architectures, and rigorous benchmarking standards. Multi-institutional datasets, richer multimodal inputs, such as complete DICOM series, reports, and clinical metadata, as well as reader studies comparing radiologists and VLLMs in realistic diagnostic workflows, will be essential. An additional important direction for future research is the evaluation of ensemble strategies combining multiple VLLMs. Aggregating predictions from complementary models may improve diagnostic robustness or mitigate individual failure modes, including hallucinations. However, such approaches also risk masking systematic errors present across models and should therefore be evaluated cautiously. Future work should systematically assess whether ensemble methods provide measurable benefits over single-model performance in realistic radiologic image interpretation tasks. Equally important are systematic assessments of hallucinations, failure modes, context dependence, and longitudinal model drift.

## 5. Conclusions

In summary, while current VLLMs demonstrate partial diagnostic capability and plausible reasoning, their overall performance, reliance on context, and high hallucination rates highlight substantial limitations in radiologic image interpretation. These findings indicate that substantial model refinement, domain-specific training, and robust validation frameworks are required to better characterize and improve VLLM performance in medical imaging tasks.

## Figures and Tables

**Table 1:** Diagnostic accuracy of visual large language models as a function of context and imaging modality. Each model interpreted 60 radiography, 60 CT, and 60 MRI cases under two conditions: uncontextualized (image only) and contextualized (image plus clinical information). Values indicate the number of correctly interpreted cases and the corresponding percentage. Bold values highlight the highest accuracy for each modality-context combination.

Model	Radiography		CT		MRI	
	Uncontextualized	Contextualized	Uncontextualized	Contextualized	Uncontextualized	Contextualized
Gemini 2.0	9/60 (15.0%)	**21/60 (35.0%)**	**20/60 (33.3%)**	**27/60 (45.0%)**	**11/60 (18.3%)**	17/60 (28.3%)
ChatGPT-4o	**12/60 (20.0%)**	20/60 (33.3%)	6/60 (10.0%)	18/60 (30.0%)	6/60 (10.0%)	**21/60 (35.0%)**
LLaVA	3/60 (5.0%)	4/60 (6.7%)	4/60 (6.7%)	10/60 (16.7%)	3/60 (5.0%)	5/60 (8.3%)
LLaVA-Med	1/60 (1.7%)	9/60 (15.0%)	3/60 (5.0%)	15/60 (25.0%)	0/60 (0.0%)	10/60 (16.7%)
Vision AI	6/60 (10.0%)	14/60 (23.3%)	8/60 (13.3%)	15/60 (25.0%)	2/60 (3.3%)	12/60 (20.0%)
Claude Sonnet 3.7	9/60 (15.0%)	18/60 (30.0%)	9/60 (15.0%)	17/60 (28.3%)	4/60 (6.7%)	13/60 (21.7%)
Perplexity AI	9/60 (15.0%)	10/60 (16.7%)	5/60 (8.3%)	16/60 (26.7%)	4/60 (6.7%)	9/60 (15.0%)

**Table 2:** Plausibility of image findings described by visual large language models in relation to their stated diagnoses. Values represent the number and percentage of plausible image descriptions out of 180 cases per model. Plausible = findings consistent with the stated diagnosis (regardless of actual presence in the image).

Model	Plausible/180 (%)
Gemini 2.0	172/180 (95.6%)
ChatGPT-4o	169/180 (93.9%)
Vision AI	161/180 (89.4%)
Claude Sonnet 3.7	158/180 (87.8%)
Perplexity AI	134/180 (74.4%)
LLaVA-Med	118/180 (65.6%)
LLaVA	91/180 (50.6%)

**Table 3:** Hallucinations included fabricated imaging findings and/or misidentifications per case of modality or anatomic region. Values represent the number and percentage of cases in which hallucinations occurred out of 180 cases per condition (360 total per model). “Uncontextualized” refers to image-only interpretation; “contextualized” refers to image interpretation supplemented with clinical information.

Model	Uncontextualized (n/180, %)	Contextualized (n/180, %)	Total (n/360, %)
Gemini 2.0	108/180 (60.0%)	78/180 (43.3%)	186/360 (51.7%)
ChatGPT-4o	152/180 (84.4%)	110/180 (61.1%)	262/360 (72.8%)
LLaVA-Med	148/180 (82.2%)	117/180 (65.0%)	265/360 (73.6%)
Vision AI	156/180 (86.7%)	129/180 (71.7%)	285/360 (79.2%)
Claude Sonnet 3.7	151/180 (83.9%)	131/180 (72.8%)	282/360 (78.3%)
LLaVA	155/180 (86.1%)	142/180 (78.9%)	297/360 (82.5%)
Perplexity AI	156/180 (86.7%)	142/180 (78.9%)	298/360 (82.8%)

## Data Availability

Data generated or analyzed during the study are available from the corresponding author upon reasonable request.

## Supplementary Materials

The following supporting information can be downloaded at: https://www.mdpi.com/article/10.3390/life16010066/s1, Text S1: Power Analysis; Table S1: Total diagnostic accuracy of visual large language models as a function of context; Table S2: Total hallucinations of visual large language models, divided into fabricated imaging findings (fabrications) and misidentification of anatomic region or imaging modality (misidentification) as a function of context.

## Abbreviations

The following abbreviations are used in this manuscript:

VLLM	Visual large language model
PACS	Picture Archiving and Communication System
DICOM	Digital Imaging and Communications in Medicine
