# Supplementary material for: Visual Large Language Models in Radiology: A Systematic Multimodel Evaluation of Diagnostic Accuracy and Hallucinations
Source: Life (Basel). 2026 Jan 1;16(1):66. doi: 10.3390/life16010066 (PMC12842777; doi:10.3390/life16010066)
Supplement: Supplementary file 1 [file life-16-00066-s001.zip › Supplementary Table S1.pdf]

life

MDPI

Article

Visual Large Language Models in Radiology: A Systematic Evaluation of Diagnostic Accuracy and Hallucinations

Marc Sebastian von der Stück *, Roman Vuskov, Simon Westfechtel, Robert Siepmann, Christiane Kuhl, Daniel Truhn and Sven Nebelung

Department of Diagnostic and Interventional Radiology, University Hospital RWTH Aachen, 52074 Aachen, Germany
* Correspondence: mvonderstuec@ukaachen.de

Supplementary Material

Supplementary Table S1. Total diagnostic accuracy of visual large language models as a function of context. Each model interpreted 180 cases either without clinical information (uncontextualized) or with clinical information (contextualized). Values represent the number of correct diagnoses and percentages. Row-wise p-values represent within-model comparisons between the two conditions. Column-wise p-values represent the global effect of model choice within each condition, based on mixed-effects logistic regression. Statistically significant values are shown in bold.

Model	Uncontextualized	Contextualized	p-Value
Gemini 2.0	**40/180 (22.2%)**	**65/180 (36.1%)**	**0.003**
ChatGPT-4o	24/180 (13.3%)	60/180 (33.3%)	**<0.001**
LLaVA	10/180 (5.6%)	19/180 (10.6%)	0.085
LLaVA-Med	4/180 (2.2%)	34/180 (18.9%)	**<0.001**
Vision AI	16/180 (8.9%)	41/180 (22.8%)	**<0.001**
Claude Sonnet 3.7	22/180 (12.2%)	48/180 (26.7%)	**<0.001**
Perplexity AI	18/180 (10.0%)	35/180 (19.4%)	**0.010**
**p-value**	**<0.001**	**<0.001**	

Academic Editors: Lisa Catarzi, Giuseppe Consorti and Guido Gabriele

Received: 2 December 2025
Revised: 29 December 2025
Accepted: 30 December 2025
Published: 1 January 2026

Copyright: © 2026 by the authors. Licensee MDPI, Basel, Switzerland. This article is an open access article distributed under the terms and conditions of the Creative Commons Attribution (CC BY) license.

 https://doi.org/10.3390/life16010066
