# Supplementary material for: Visual Large Language Models in Radiology: A Systematic Multimodel Evaluation of Diagnostic Accuracy and Hallucinations
Source: Life (Basel). 2026 Jan 1;16(1):66. doi: 10.3390/life16010066 (PMC12842777; doi:10.3390/life16010066)
Supplement: Supplementary file 1 [file life-16-00066-s001.zip › Supplementary Table S2.pdf]

life

Article

Visual Large Language Models in Radiology: A Systematic Evaluation of Diagnostic Accuracy and Hallucinations

Marc Sebastian von der Stück *, Roman Vuskov, Simon Westfechtel, Robert Siepmann, Christiane Kuhl, Daniel Truhn and Sven Nebelung

Department of Diagnostic and Interventional Radiology, University Hospital RWTH Aachen, 52074 Aachen, Germany
* Correspondence: mvonderstuec@ukaachen.de

Supplementary Material

Supplementary Table S2. Total hallucinations of visual large language models, divided into fabricated imaging findings (fabrications) and misidentification of anatomic region or imaging modality (misidentification) as a function of context. Each model interpreted 180 cases either without clinical information (uncontextualized) or with clinical information (contextualized). Values represent the number of cases and percentages.

Model	Uncontextualized (n=180)		Contextualized (n=180)		Total (n=360)	
	Fabrications	Misidentifications	Fabrications	Misidentifications	Fabrications	Misidentifications
Gemini 2.0	108 (60.0%)	10 (5.6%)	78 (43.3%)	5 (2.8%)	186 (51.7%)	15 (4.2%)
ChatGPT-4o	152 (84.4%)	19 (10.6%)	110 (61.1%)	3 (1.7%)	262 (72.8%)	22 (6.1%)
LLaVA-Med	148 (82.2%)	35 (19.4%)	117 (65.0%)	19 (10.6%)	265 (73.6%)	54 (15.0%)
Vision AI	156 (86.7%)	7 (3.9%)	129 (71.7%)	3 (1.7%)	285 (79.2%)	10 (2.8%)
Claude Sonnet 3.7	151 (83.9%)	4 (2.2%)	131 (72.8%)	1 (0.6%)	282 (78.3%)	5 (1.4%)
LLaVA	155 (86.1%)	9 (5.0%)	142 (78.9%)	0 (0.0%)	297 (82.5%)	9 (2.5%)
Perplexity AI	156 (86.7%)	1 (0.6%)	142 (78.9%)	0 (0.0%)	298 (82.8%)	1 (0.3%)

Academic Editors: Lisa Catarzi, Giuseppe Consorti and Guido Gabriele

Received: 2 December 2025
Revised: 29 December 2025
Accepted: 30 December 2025
Published: 1 January 2026

Copyright: © 2026 by the authors. Licensee MDPI, Basel, Switzerland. This article is an open access article distributed under the terms and conditions of the Creative Commons Attribution (CC BY) license.
