# Supplementary material for: Visual Large Language Models in Radiology: A Systematic Multimodel Evaluation of Diagnostic Accuracy and Hallucinations
Source: Life (Basel). 2026 Jan 1;16(1):66. doi: 10.3390/life16010066 (PMC12842777; doi:10.3390/life16010066)
Supplement: Supplementary file 1 [file life-16-00066-s001.zip › Supplementary Text S1.pdf]

*life*

MDPI

*Article*

# Visual Large Language Models in Radiology: A Systematic Evaluation of Diagnostic Accuracy and Hallucinations

**Marc Sebastian von der Stück *, Roman Vuskov, Simon Westfechtel, Robert Siepmann, Christiane Kuhl, Daniel Truhn and Sven Nebelung**

Department of Diagnostic and Interventional Radiology, University Hospital RWTH Aachen, 52074 Aachen, Germany
* Correspondence: mvonderstuec@ukaachen.de

## Supplementary Material

*Supplementary Text S1: Power Analysis*

A priori sample size calculations were performed to ensure sufficient power to detect clinically relevant differences in accuracy. Given the observed overall mean accuracy of 17.3% (range, 8.1%–29.2%), we conservatively assumed a baseline accuracy of 20% and aimed to detect an absolute improvement of 10% (i.e., 20% vs. 30%). Using a two-sided test at $\alpha = 0.05$ and a desired power of 0.80, the required sample size per group was calculated as approximately 292 paired observations (Cohen's $h = 0.23$).

In our study, each of the seven models was evaluated on 180 radiologic images, both with and without context, resulting in 360 paired observations per model and a total of 2,520 assessments across all models. Thus, the available sample size substantially exceeded the minimum requirement.

Academic Editors: Lisa Catarzi, Giuseppe Consorti and Guido Gabriele

Received: 2 December 2025
Revised: 29 December 2025
Accepted: 30 December 2025
Published: 1 January 2026

**Copyright:** © 2026 by the authors. Licensee MDPI, Basel, Switzerland. This article is an open access article distributed under the terms and conditions of the Creative Commons Attribution (CC BY) license.
